# Real-world safety profile of elexacaftor/tezacaftor/ivacaftor: a disproportionality analysis using the U.S. FDA adverse event reporting system

**DOI:** 10.3389/fphar.2025.1531514

**Published:** 2025-03-12

**Authors:** Chengyu Zhu, Zhiwei Cui, Tingting Liu, Siyu Lou, Linmei Zhou, Junyou Chen, Ruizhen Zhao, Li Wang, Yingyong Ou, Fan Zou

**Affiliations:** ^1^ Department of Respiratory and Critical Care Medicine, Affiliated Hospital of Zunyi Medical University, Zunyi, China; ^2^ Department of Obstetrics and Gynecology, The First Affiliated Hospital of Xi’an Jiaotong University, Xi’an, China

**Keywords:** CFTR modulator, pharmacovigilance, FAERS, real world analysis, adverse drug events

## Abstract

**Background:**

Elexacaftor/Tezacaftor/Ivacaftor (ETI) has demonstrated significant efficacy in enhancing clinical outcomes for patients with cystic fibrosis (CF). Despite this, comprehensive post-marketing assessments of its adverse drug events (ADEs) remain insufficient. This study aims to analyze the ADEs associated with ETI using the U.S. FDA Adverse Event Reporting System (FAERS).

**Methods:**

We conducted a pharmacovigilance analysis utilizing FAERS data from Q4 2019 to Q3 2024. Reports of ADEs related to ETI were extracted, and disproportionality analyses—including Reporting Odds Ratio (ROR), Proportional Reporting Ratio (PRR), Bayesian Confidence Propagation Neural Network (BCPNN), and Multi-item Gamma Poisson Shrinker (MGPS)—were employed to evaluate signal strength. Additionally, a time-to-onset (TTO) analysis was performed.

**Results:**

A total of 28,366 ETI-related ADEs were identified, spanning 27 organ systems. We identified 322 positive signals, with signals consistent with the drug label including headache (702 cases, ROR 2.75), infective pulmonary exacerbation of CF (691 cases, ROR 384.24), rash (538 cases, ROR 2.72), and cough (507 cases, ROR 3.79). Unexpected signals were also noted, such as anxiety (494 cases, ROR 4.16), depression (364 cases, ROR 4.59), insomnia (281 cases ROR 2.83), nephrolithiasis (79 cases, ROR 3.63) and perinatal depression (4 cases, ROR 13.59). The TTO analysis indicated that the median onset of ADEs was 70 days, with 37.08% occurring within the first month. Subgroup analyses revealed that females exhibited a higher reporting rank for mental disorder and constipation, whereas in males, they were insomnia, abdominal pain, and nasopharyngitis.

**Conclusion:**

This study highlights both recognized and unexpected ADEs associated with ETI, underscoring the necessity for ongoing monitoring, particularly concerning psychiatric conditions. The subgroup analysis suggests a need for personalized treatment strategies to optimize patient care.

## 1 Introduction

Cystic fibrosis (CF) is an autosomal recessive genetic disorder caused by mutations in the gene encoding the CF transmembrane conductance regulator (CFTR) protein (Fajac and Burgel, 2023). Approximately 90% of CF patients carry at least one copy of the Phe508del CFTR mutation (Fanen et al., 2014). Dysfunction of CFTR leads to impaired chloride and bicarbonate transport in epithelial cells, resulting in a multi-organ disease that primarily affects the respiratory and digestive systems. Globally, there are about 89,000 diagnosed cases of CF, with approximately 1,000 new cases reported each year.

Current pulmonary therapies for CF include mucolytics, anti-inflammatories, and antibiotics. Recently, four small-molecule therapies, collectively referred to as CFTR modulators, have been approved by regulatory authorities to enhance CFTR production and/or function (Lopes-Pacheco, 2019). Among these, the triple combination of Elexacaftor/Tezacaftor/Ivacaftor (ETI) has emerged as a novel CFTR modulator that significantly improves various clinical outcomes in CF patients. In a Phase 2 trial involving individuals with the heterozygous Phe508del mutation, ETI administration led to a 13.8-point increase in the percentage of predicted forced expiratory volume in 1 s (FEV1) at 4 weeks and a 14.3-point increase at 24 weeks, alongside a 63% reduction in the rate of pulmonary exacerbations. Additionally, patients reported enhanced quality of life regarding respiratory symptoms and exhibited a significant decrease in sweat chloride concentration, averaging 41.8 mmol per liter lower than baseline (Middleton et al., 2019). This triple combination therapy was first approved in the United States in 2019 for patients aged 12 years and older and became available for children aged 6 years and older in 2021 (Hoy, 2019).

Despite the demonstrated efficacy of ETI in clinical practice, there has been a gradual increase in reports of associated adverse drug events (ADEs). A Phase 3 study by Zemanick et al. evaluated the safety of ETI in children aged 6–11 years. Among the 66 children included in the study, ADEs were reported by 98.5% of participants. Most events were categorized as mild (54.5%) or moderate (42.4%) in severity and were generally consistent with manifestations commonly observed in CF or typical childhood infections. The most frequently reported ADEs included cough (42.4%), headache (24.2%), pyrexia (24.2%), oropharyngeal pain (18.2%), and upper respiratory tract infection (16.7%). Notably, one child experienced an erythematous rash following the first dose of ETI which necessitated discontinuation of the study drug (Zemanick et al., 2021).

Although clinical trials have primarily documented the efficacy and safety of ETI, the sample sizes are limited and the studies have specific selection criteria. ICSR (Individual Case Safety Reports) databases, such as the U.S. FDA Adverse Event Reporting System (FAERS), and disproportionality analysis are powerful tools in pharmacovigilance and drug safety. When disproportionality analysis is applied to ICSR databases, it helps identify signals for rare adverse events by comparing the observed number of reports for a specific drug-adverse event pair with the expected number (Bate and Evans, 2009). This approach is particularly useful in addressing gaps in the knowledge of a drug’s safety profile in real-world settings. This pharmacovigilance analysis represents the first comprehensive assessment of the post-marketing safety of ETI using the U.S. FAERS database. The primary objective is to provide critical insights for clinical surveillance and to identify potential hazards associated with ETI.

## 2 Methods

### 2.1 Data collection and deduplication

The FAERS database, one of the largest publicly available databases on ADEs, includes more than 9 million individual reports of drug-related adverse events submitted by healthcare professionals and industry, offering researchers raw data directly from the FDA website (https://fs.fda.gov/extensions/FPDQDE-FAERS/FPD-QDE-FAERS.html) (van Hasselt et al., 2020). The FAERS database is updated quarterly and comprises seven datasets: demographic and administrative information (DEMO), drug information (DRUG), adverse drug reaction information (REAC), patient outcome information (OUCT), reporting source (RPSR), drug treatment start and end dates (THER), and drug administration indications (INDI). Following the approval of ETI by the FDA on 21 October 2019, we extracted data spanning from the fourth quarter of 2019 (Q4 2019) to the third quarter of 2024 (Q3 2024).

Given the nature of data updates, duplicate reports in FAERS are unavoidable. To enhance the reliability of our findings, we adhere strictly to the U.S. FDA’s official guideline to identify and remove duplicates. In the DEMO file, we selected the PRIMARYIDs, CASEIDs, and FDA_DTs, subsequently sorting them by CASEIDs, FDA_DTs, and PRIMARYIDs. If multiple entries shared the same CASEID, the most recent FDA_DT was retained. In cases where both the CASEID and FDA_DT were identical, the entry with the higher PRIMARYID was selected (Wan et al., 2022). This criterion ensures the removal of duplicate reports from different individuals and institutions, as each case report is assigned a unique PRIMARYID, with higher values indicating more recently submitted reports (Cao et al., 2024; Gao et al., 2025). Furthermore, starting from the first quarter of 2019, each quarterly data package includes a deletion report list. Following the deduplication process, reports are deleted based on the CASEID listed in the deletion report. These steps effectively eliminate redundant entries, thereby ensuring the robustness of subsequent analyses. Since FAERS does not utilize a uniform drug coding system, we identified ETI-associated ADE reports using both generic and brand names, including “TRIKAFTA” and “ELEXACAFTOR IVACAFTOR TEZACAFTOR.” To improve the accuracy of our results and mitigate the potential impact of concomitant medications, we retained only those ADE reports classified as primary suspect (PS). ADEs were coded using the system organ class (SOC) terminology based on the top-level classification of the Medical Dictionary for Regulatory Activities (MedDRA, version 27.0) (Brown, 2004). We extracted all preferred terms (PTs) from MedDRA and excluded terms that appeared fewer than three times in FAERS. Following preprocessing, we screened a total of 28,366 preferred terms related to ETI.

### 2.2 Disproportionality analysis

Disproportionality analysis is a key method used in pharmacovigilance to identify potential causal relationships between drugs and adverse events (Montastruc et al., 2011). By comparing the observed number of reports to the expected number for each drug-adverse event pair, it helps detect signals that may indicate an increased risk of adverse reactions, providing valuable insights for post-market safety monitoring (de Boer, 2011). Disproportionality analysis methods, including the Reporting Odds Ratio (ROR) (van Puijenbroek et al., 2002a), Proportional Reporting Ratio (PRR) (Evans et al., 2001), Bayesian Confidence Propagation Neural Network (BCPNN) (Bate et al., 1998a), and Multiple Gamma Poisson Shrinkage (MGPS) (Szarfman et al., 2002), are employed to assess signal strength for ADEs (Gu et al., 2024). The ROR and PRR algorithms are known for their high sensitivity and ease of calculation. However, these methods have a higher likelihood of generating false positives, particularly when the number of reported adverse events is low (van Puijenbroek et al., 2002b). In contrast, BCPNN and MGPS demonstrate greater stability with limited report numbers, thereby reducing the risk of false positives and effectively handling high-dimensional pattern recognition. Nonetheless, these algorithms can be computationally complex and slow in signal detection (Bate et al., 1998b).

**FIGURE 1:**
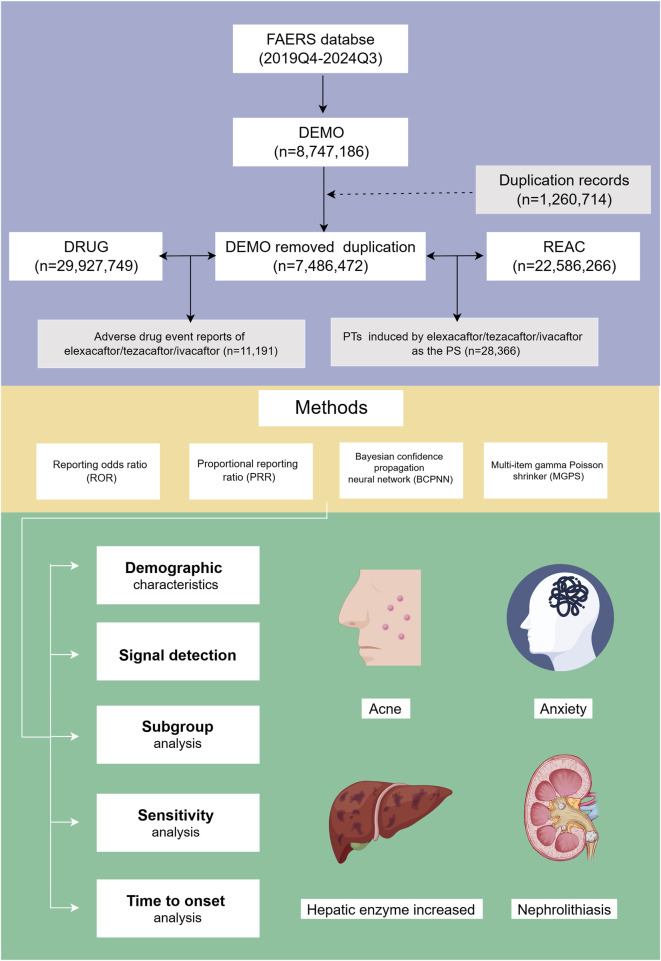
Flowchart illustrating the study design and key findings. FAERS: Food and Drug Administration Adverse Event Reporting System; PTs, preferred terms; PS, primary suspect; Q3, third quarter; Q4, fourth quarter.

In this study, we employed multiple algorithms simultaneously to capitalize on the strengths of each, thereby broadening the detection scope and enabling a multifaceted review of results for more comprehensive and reliable safety signal detection (Cui et al., 2024). All analyses were conducted based on a 2 × 2 contingency table (as detailed in Table 1). To enhance the reliability of our findings, we considered only PTs that were identified as positive signals by all four algorithms. Additionally, we excluded ADEs related to drug indications to ensure clarity in our statements. Unexpected signals not listed on the drug label were deemed significant. The workflow and main results of our analysis are illustrated in Figure 1.

**TABLE 1:** Methods, formulas, and thresholds for calculating Reporting Odds Ratio (ROR), Proportional Reporting Ratio (PRR), Bayesian Confidence Propagation Neural Network (BCPNN), and Empirical Bayesian Geometric Mean (EBGM). Variable “a” represents the number of individuals experiencing expected adverse events following exposure to elexacaftor/tezacaftor/ivacaftor (ETI), variable “b” represents the number of individuals experiencing non-target adverse events following ETI exposure, variable “c” represents the number of individuals experiencing target adverse events following non-ETI exposure, and variable “d” represents the number of individuals experiencing non-target adverse events following non-ETI exposure. Abbreviations: 95% CI: 95% confidence interval; χ^2^: chi-squared; IC: information component; IC025: Information Component 2.5th percentile; E (IC): expected IC; V(IC): variance of IC; EBGM05: Empirical Bayes Geometric Mean 5th percentile.

	Target adverse drug event	Non-target adverse drug event	Sums
elexacaftor/tezacaftor/ivacaftor (ETI)	a	b	a+b
Non-ETI	c	d	c + d
Total	a+c	b + d	a+b + c + d

Methods	Formula	Threshold
ROR	$ROR=\frac{a/c}{b/d}$	a≥3
	$SE\left(lnROR\right)=\sqrt{\left(\frac{1}{a}+\frac{1}{b}+\frac{1}{c}+\frac{1}{d}\right)}$	
	$95\%\text{CI}=e^{\ln\left(\mathrm{ROR}\right)\pm 1.96\text{se}}$	$95\%\text{CI}\left(\text{lower limit}\right)>1$
PRR	$\mathrm{PRR}=\frac{a/\left(a+b\right)}{c/\left(c+d\right)}$	a≥3
	$\chi^{2}=\left[{\left(\text{ad}-\text{bc}\right)}^{2}\right]\left(a+b+c+d\right)/\left[\left(a+b\right)\;\left(c+d\right)\;\left(a+c\right)\;\left(b+d\right)\right]$	χ2≥4
BCPNN	$\mathrm{IC}=\log_{2}\frac{p\left(x,y\right)}{p\left(x\right)p\left(y\right)}=\log_{2}\frac{a\left(a+b+c+d\right)}{\left(a+b\right)\left(a+c\right)}$	IC025>0
	$E\left(\mathrm{IC}\right)=\log_{2}\frac{\left(a+\gamma 11\right)\left(a+b+c+d+\alpha \right)\left(a+b+c+d+\beta \right)}{\left(a+b+c+d+\gamma \right)\left(a+b+\alpha 1\right)\left(a+c+\beta 1\right)}$	
	$V\left(\mathrm{IC}\right)=\frac{1}{{\left(\ln2\right)}^{2}}\left[\frac{\left(a+b+c+d\right)-a+\gamma -\gamma 11}{\left(a+\gamma 11\right)\left(1+a+b+c+d+\gamma \right)}+\frac{\left(a+b+c+d\right)-\left(a+b\right)+a-\alpha 1}{\left(a+b+\alpha 1\right)\left(1+a+b+c+d+\alpha \right)}+\frac{\left(a+b+c+d+\alpha \right)-\left(a+c\right)+\beta -\beta 1}{\left(a+b+\beta 1\right)\left(1+a+b+c+d+\beta \right)}\right]$	
	$\gamma =\gamma 11\frac{\left(a+b+c+d+\alpha \right)\left(a+b+c+d+\beta \right)}{\left(a+b+\alpha 1\right)\left(a+c+\beta 1\right)}$	
	$\mathrm{IC}-2\mathrm{SD}=E\left(\mathrm{IC}\right)-2\sqrt{V\left(\mathrm{IC}\right)}$	
EBGM	$\mathrm{EBGM}=\frac{a\left(a+b+c+d\right)}{\left(a+c\right)\left(a+b\right)}$	EBGM05>2
	$\mathrm{SE}\left(\mathrm{lnEBGM}\right)=\sqrt{\left(\frac{1}{a}+\frac{1}{b}+\frac{1}{c}+\frac{1}{d}\right)}$	
	$95\%\text{CI}=e^{\ln\left(\mathrm{EBGM}\right)\pm 1.96\text{se}}$	

### 2.3 Time-to-onset (TTO) analysis

In this study, we defined the time-to-onset (TTO) of drug-related ADEs as the interval between the onset date of the ADE (EVENT_DT) in the demographic file (DEMO) and the start date of ETI administration (START_DT) in the therapy file (THER). Cases with inaccurate or missing data, as well as instances where the ADE onset date preceded the drug administration date, were excluded from analysis. We employed the Weibull distribution to estimate the variation in the risk of ADEs over time (Zhang W. et al., 2024). We also employed the Kaplan-Meier method to illustrate the cumulative incidence curves of ETI-related ADEs across subgroups and conducted intergroup comparisons using the log-rank test (Wang et al., 2024). For TTO analyses at the PT level, differences between two groups were assessed using the Wilcoxon rank-sum test, while differences among multiple groups were evaluated using the Kruskal-Wallis test (Zou et al., 2024). A *P*-value less than 0.05 was considered statistically significant.

### 2.4 Analysis software

All data processing and statistical analyses were conducted using Microsoft Excel 2019 and R software (version 4.2.1).

## 3 Results

### 3.1 Descriptive analysis

**FIGURE 2:**
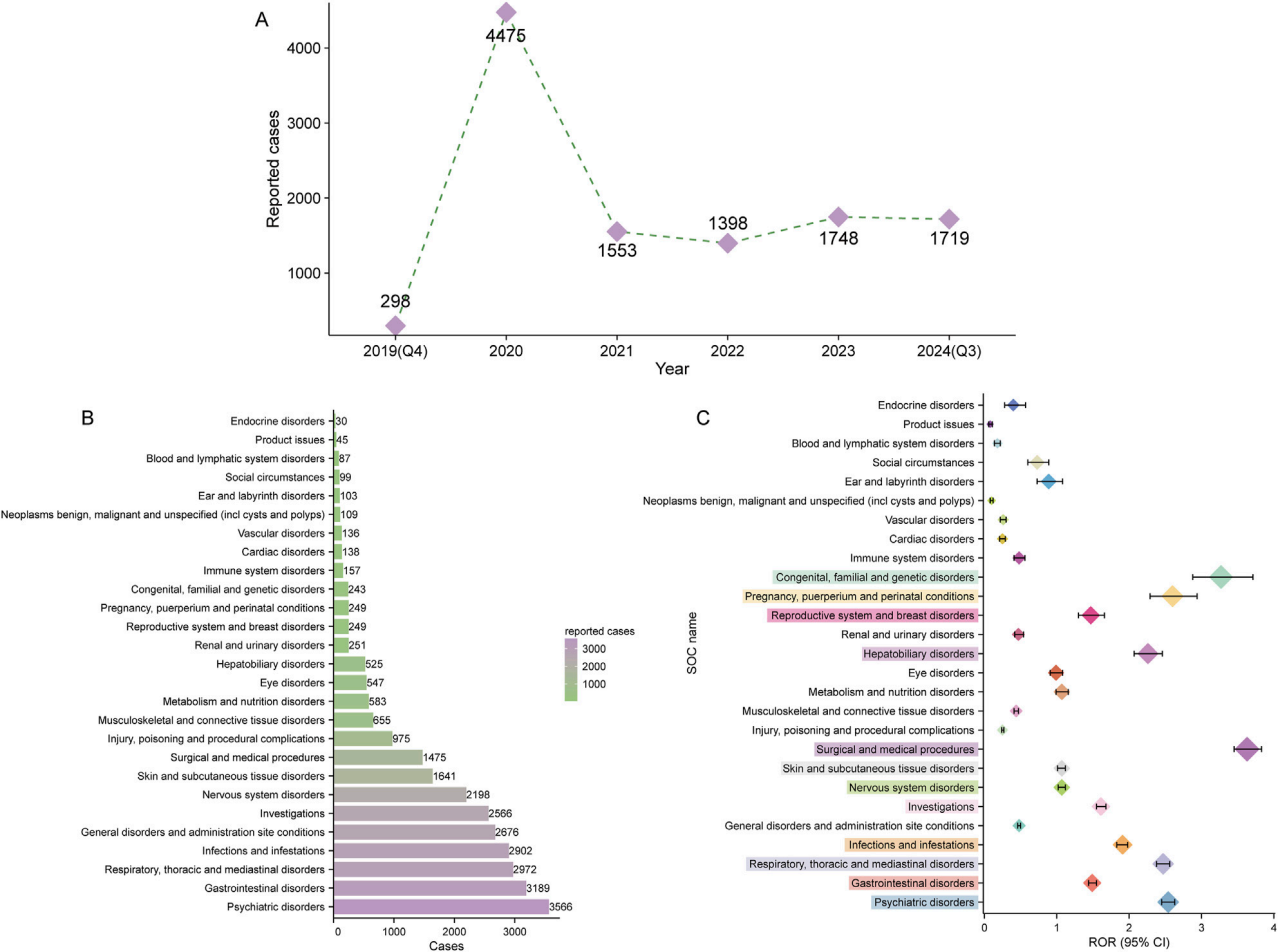
Signal detection at the system organ class (SOC) level. **(A)** Distribution of adverse drug events (ADEs) associated with elexacaftor/tezacaftor/ivacaftor from the fourth quarter of 2019 (Q4 2019) to the third quarter of 2024 (Q3 2024). **(B)** Bar chart displaying the number of reported ADEs at each SOC level. **(C)** Signal detection at the SOC level, with reporting odds ratios (RORs) and their 95% confidence intervals visualized. The SOCs that meet the threshold for the ROR method are highlighted in prominent colors.

After data cleansing and deduplication, a total of 11,191 ETI-associated ADE reports were identified, encompassing 28,366 PTs attributed to ETI as the PS (Figure 1). The detailed clinical characteristics of these ETI-related ADEs are summarized in Table 2. As shown in Figure 2A, the ADEs associated with ETI peaked in 2020 and were recorded from the fourth quarter of 2019 to the third quarter of 2024.

**TABLE 2:** Demographic characteristics of ADEs reported in the FAERS database with elexacaftor/tezacaftor/ivacaftor as the primary suspect drug. Abbreviations: US, United States; GB, Great Britain; FR, France; CA, Canada; DE, Germany; HO, hospitalization; LT, life-threatening; DS, disability; DE, death; OT, other serious outcomes; RI, required intervention; CA, congenital anomaly.

Characteristics	Case number	Case proportion, %
Sex		
Female	5584	49.9%
Male	4232	37.8%
Unknown	1375	12.3%
Age (years)		
<2	30	0.3%
2–5	133	1.2%
6–11	358	3.2%
12–17	972	8.7%
18–65	3576	32.0%
>65	131	1.1%
Unknown	5991	53.5%
Weight (kg)		
<50	758	6.8%
50–100	1994	17.8%
>100	52	0.5%
Unknown	8387	74.9%
Reported Countries (top five)		
US	8668	77.5%
GB	1708	15.3%
FR	221	2.0%
CA	97	0.9%
DE	86	0.8%
Reported person		
Health professionals	5779	51.6%
Consumer	5404	48.3%
Unknown	8	0.1%
Outcome		
HO	3278	29.3%
LT	64	0.6%
DS	23	0.2%
DE	268	2.4%
OT	1266	11.3%
RI	5	0.0%
CA	37	0.3%
Unknown	6250	55.8%
Indication (top three)		
Cystic fibrosis	11151	99.6%
Infective pulmonary exacerbation of cystic fibrosis	9	0.1%
Cystic fibrosis lung	3	0.0%

### 3.2 Disproportionality analysis

ETI-related ADEs spanned 27 SOCs. Table 3 presents the number and signal strength of these ADEs at the SOC level, which are also visualized in Figure 2B. Using the four disproportionality analysis methods, we identified 12 positive signals at the SOC level (Figure 2C). The top two SOCs based on ROR values were surgical and medical procedures (1,475 cases, ROR 3.63 [95% CI 3.45–3.83]) and congenital, familial, and genetic disorders (243 cases, ROR 3.27 [95% CI 2.88–3.71]). Notably, six SOCs including psychiatric disorders (ROR: 2.54, PRR: 2.35, EBGM05: 2.28, IC025: 1.18), respiratory, thoracic and mediastinal disorders (ROR: 2.47, PRR: 2.32, EBGM05: 2.24, IC025: 1.15), surgical and medical procedures (ROR: 3.63, PRR: 3.49, EBGM05: 3.33, IC025: 1.72), hepatobiliary disorders (ROR: 2.26, PRR: 2.23, EBGM05: 2.07, IC025: 1.03), pregnancy, puerperium and perinatal conditions (ROR: 2.60, PRR: 2.58, EBGM05: 2.32, IC025: 1.18), and congenital, familial and genetic disorders (ROR: 3.27, PRR: 3.25, EBGM05: 2.92, IC025: 1.51) simultaneously met the thresholds of all four disproportionality analysis methods, indicating a statistically significant association between ETI use and ADEs under the organ system level.

**TABLE 3:** Signal strength of ADE reports for elexacaftor/tezacaftor/ivacaftor at the SOC level in the FAERS database. ADE, adverse drug event; SOC, system organ class.

System Organ Class	Cases	ROR (95% CI)	PRR (χ2)	EBGM (EBGM05)	IC (IC025)
Psychiatric disorders	3566	2.54 (2.45–2.63)	2.35 (2908.29)	2.34 (2.28)	1.23 (1.18)
Gastrointestinal disorders	3189	1.49 (1.44–1.55)	1.43 (455.27)	1.43 (1.39)	0.52 (0.47)
Respiratory, thoracic and mediastinal disorders	2972	2.47 (2.38-2.56)	2.32 (2319.36)	2.31 (2.24)	1.21 (1.15)
Infections and infestations	2902	1.91 (1.83-1.98)	1.81 (1118.07)	1.81 (1.75)	0.86 (0.8)
General disorders and administration site conditions	2676	0.48 (0.46-0.5)	0.53 (1339.63)	0.53 (0.51)	−0.91 (−0.97)
Investigations	2566	1.61 (1.55–1.68)	1.56 (542.56)	1.56 (1.5)	0.64 (0.58)
Nervous system disorders	2198	1.07 (1.02–1.12)	1.06 (9.27)	1.06 (1.03)	0.09 (0.03)
Skin and subcutaneous tissue disorders	1641	1.07 (1.01–1.12)	1.06 (6.31)	1.06 (1.02)	0.09 (0.01)
Surgical and medical procedures	1475	3.63 (3.45-3.83)	3.49 (2655.14)	3.48 (3.33)	1.8 (1.72)
Injury, poisoning and procedural complications	975	0.25 (0.24–0.27)	0.28 (2107.09)	0.28 (0.26)	−1.85 (−1.95)
Musculoskeletal and connective tissue disorders	655	0.44 (0.41–0.47)	0.45 (460.88)	0.45 (0.42)	−1.15 (−1.26)
Metabolism and nutrition disorders	583	1.07 (0.99–1.16)	1.07 (2.77)	1.07 (1)	0.1 (−0.02)
Eye disorders	547	0.99 (0.91–1.08)	0.99 (0.05)	0.99 (0.92)	−0.01 (−0.14)
Hepatobiliary disorders	525	2.26 (2.07–2.46)	2.23 (359.47)	2.23 (2.07)	1.16 (1.03)
Renal and urinary disorders	251	0.47 (0.42–0.54)	0.48 (145.99)	0.48 (0.43)	−1.07 (−1.25)
Reproductive system and breast disorders	249	1.47 (1.3–1.66)	1.47 (36.92)	1.46 (1.32)	0.55 (0.37)
Pregnancy, puerperium and perinatal conditions	249	2.6 (2.29–2.94)	2.58 (241.24)	2.58 (2.32)	1.37 (1.18)
Congenital, familial and genetic disorders	243	3.27 (2.88–3.71)	3.25 (378.4)	3.24 (2.92)	1.7 (1.51)
Immune system disorders	157	0.48 (0.41–0.56)	0.48 (89.49)	0.48 (0.42)	−1.06 (−1.29)
Cardiac disorders	138	0.25 (0.21–0.29)	0.25 (317.96)	0.25 (0.22)	−2 (−2.25)
Vascular disorders	136	0.26 (0.22–0.3)	0.26 (291.93)	0.26 (0.23)	−1.94 (−2.19)
Neoplasms benign, malignant and unspecified (incl cysts and polyps)	109	0.1 (0.08–0.12)	0.1 (919.79)	0.1 (0.09)	−3.32 (−3.6)
Ear and labyrinth disorders	103	0.89 (0.73–1.08)	0.89 (1.49)	0.89 (0.75)	−0.17 (−0.46)
Social circumstances	99	0.73 (0.6–0.89)	0.73 (9.88)	0.73 (0.62)	−0.45 (−0.74)
Blood and lymphatic system disorders	87	0.18 (0.14–0.22)	0.18 (335.49)	0.18 (0.15)	−2.49 (−2.8)
Product issues	45	0.08 (0.06–0.11)	0.08 (463.96)	0.08 (0.07)	−3.59 (−4.01)
Endocrine disorders	30	0.4 (0.28–0.57)	0.4 (27.45)	0.4 (0.29)	−1.33 (−1.85)

**FIGURE 3:**
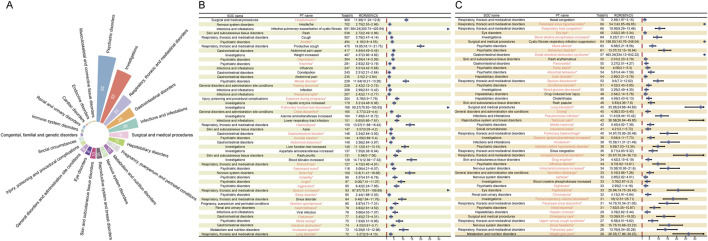
Signal detection at the preferred term (PT) level. **(A)** SOC attribution and the number of PTs (n = 322) that simultaneously meet the criteria of the four methods with positive signal values. **(B–C)** PT entries that satisfy the four methods and have a case count of at least 25, presented in descending order of case number. The forest plot displays the ROR values and their 95% confidence intervals for each PT. Asterisks (*) denote unexpected signals not listed in the drug label.

After screening and excluding signals unrelated to drug therapy, influenced by primary pathology, or associated with potential indications, we identified 322 positive signals across 22 SOCs. Figure 3A illustrates the distribution and number of positive PT signals across different SOCs. The majority of positive PTs were in the categories of psychiatric disorders (n = 62), investigations (n = 52), and respiratory, thoracic, and mediastinal disorders (n = 33). The PTs reported with at least 25 cases are shown in Figures 3B, C, with the most frequently reported being hospitalization (960 cases), headache (702 cases), infective pulmonary exacerbation of CF (691 cases), rash (538 cases), cough (507 cases), and anxiety (494 cases). Most of these signals are consistent with adverse reactions already listed in the drug label. Notably, our analysis revealed several unexpected ADEs, including anxiety (494 cases, ROR 4.16 [95% CI 3.80–4.55]), depression (364 cases, ROR 4.59 [95% CI 4.14–5.09]), insomnia (281 cases, ROR 2.83 [95% CI 2.52–3.19]), nephrolithiasis (79 cases, ROR 3.63 [95% CI 2.91–4.52]), pancreatitis (55 cases, ROR 3.27 [95% CI 2.51–4.27]), and perinatal depression (4 cases, ROR 13.59 [95% CI 5.06–36.51]). The detailed signal values for all 322 positive signals are fully provided in Supplementary Table S1.

### 3.3 Subgroup analysis

**FIGURE 4:**
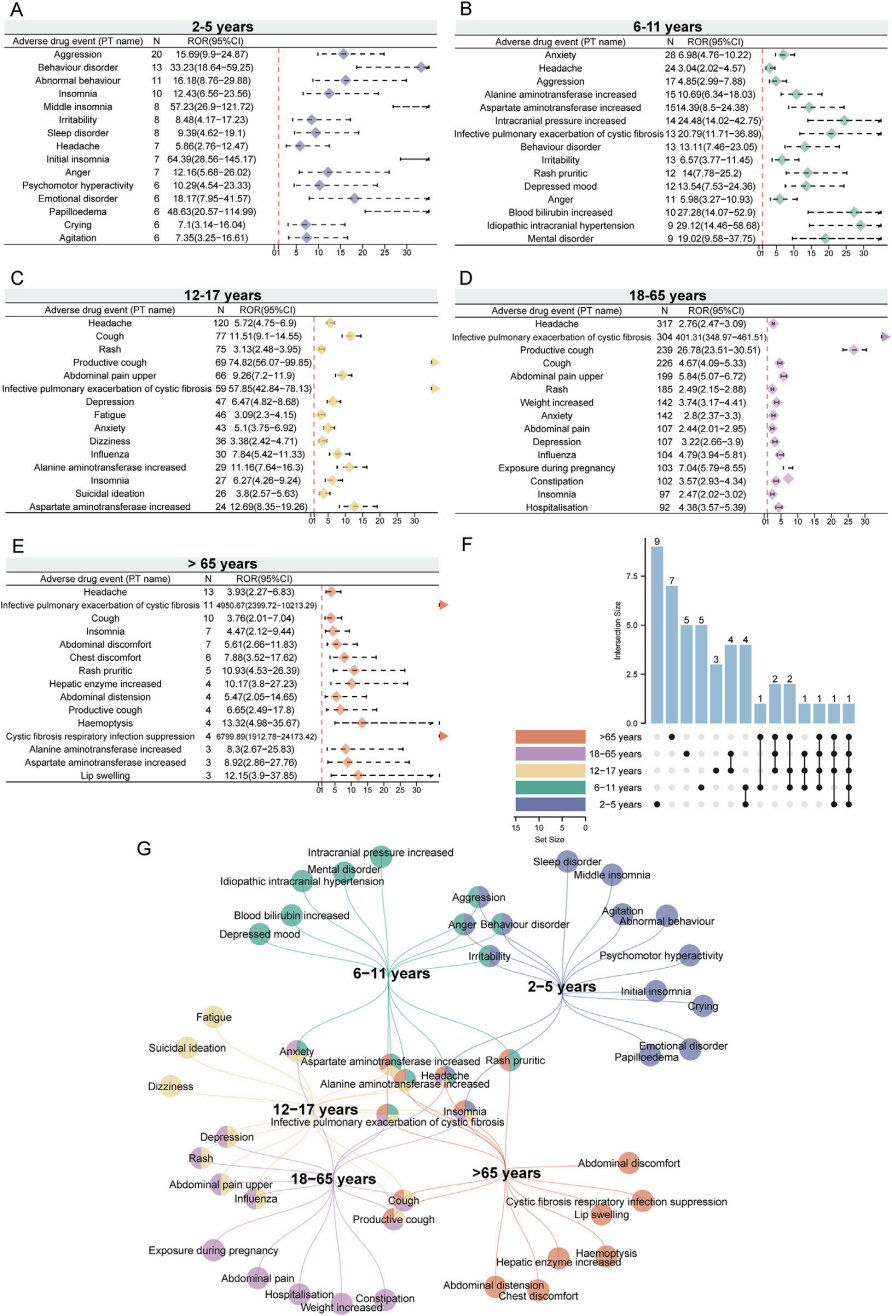
Age-related subgroup analysis of elexacaftor/tezacaftor/ivacaftor-related ADEs. We listed the top 15 PTs based on case numbers, with threshold values calculated using four different methods. The ROR values and 95% confidence intervals for the age subgroups of 2–5 years **(A)**, 6–11 years **(B)**, 12–17 years **(C)**, 18–65 years **(D)**, and over 65 years **(E)** were visualized using forest plots. **(F)** The upset plot displays the number of PT intersections across different age subgroups. **(G)** The network Venn diagram illustrates the detailed intersections of PTs across different age subgroups.

Given that variables such as age, sex, and weight may significantly influence drug-related adverse events (Shu et al., 2022; Stader and Marzolini, 2022), we performed disproportionality analysis in different subgroups based on these variables to minimize their potential confounding effects on the overall results. Considering the age-specific indications for ETI, we categorized the reporters into different age subgroups (2–5 years, 6–11 years, 12–17 years, 18–65 years, and over 65 years) and conducted the analysis. We present the top 15 positive signals based on case numbers within each age subgroup. Interestingly, despite the overlap of positive signals across different age subgroups, some positive signals appear with higher reporting ranks in certain subgroups. This phenomenon is more pronounced in the 2–5 years and over 65 years subgroups (Figure 4F). In the 2–5 years subgroup, the unique signals with higher reporting ranks include sleep disorder, middle insomnia, agitation, and emotional disorder (Figure 4A), while in the over 65 years subgroup, they include abdominal discomfort, CF respiratory infection suppression, hemoptysis, and hepatic enzyme increased (Figure 4E). Additionally, specific signals identified in other subgroups are also noteworthy, such as depressed mood and increased blood bilirubin in the 6–11 years subgroup (Figure 4B), suicidal ideation and dizziness in the 12–17 years subgroup (Figure 4C), and weight increased and constipation in the 18–65 years subgroup (Figure 4D). Interestingly, headache appears across all age groups (Figure 4F). Other signals that appear in most age groups include insomnia and infective pulmonary exacerbation of CF (Figure 4G). These findings suggest that ETI-related ADEs may vary across different age groups. Therefore, while focusing on commonly reported signals across age groups, particular attention should also be paid to the specific signals of each subgroup.

The signals were also subjected to subgroup analyses based on sex (Supplementary Figures S1A, S1B) and weight (Supplementary Figures S1D, S1E) to facilitate a comparative analysis of similarities and differences. In the sex subgroup analysis, several overlapping signals were identified, including weight increased, headache, depression, and influenza (Supplementary Figure S1C). Furthermore, in males, we found insomnia, abdominal pain, and nasopharyngitis had higher reporting ranks (Supplementary Figure S1A), while in females, mental disorder and constipation were more prominent (Supplementary Figure S1B). In the weight-based subgroup analysis, positive signals identified exclusively in the low-weight group (weight <50 kg) included elevated transaminases, irritability, and pruritic rash (Supplementary Figure S1D), while specific signals in the medium-weight group (weight between 50–100 kg) included weight increased, insomnia, and chest discomfort (Supplementary Figure S1E). The overlapping signals in the weight subgroups are presented in Supplementary Figure S1F. It is important to note that some signals identified in specific subgroups were not detected in the overall analysis (such as dizziness in the 12–17 years subgroup, and lip swelling in the >65 years subgroup), highlighting the significance of subgroup analyses. However, given the differences in reporting numbers across subgroups, caution is needed when interpreting these findings.

### 3.4 Sensitivity analysis

Although ETI is not recommended for concomitant use with other drugs in routine clinical practice, we identified some reports of concomitant drug use in the FAERS data. To exclude the potential impact of concomitant medications on the results, we excluded reports involving the use of other drugs. After excluding these reports, we identified 4,777 reports involving 9,561 adverse events. Using four methods of disproportionality analysis, our sensitivity analysis identified 182 positive signals. Persistent potential adverse reactions included rash, hospitalization, headache, anxiety, depression, insomnia, acne, hepatic enzyme increased, rash pruritic, and nephrolithiasis, among others (Supplementary Table S2).

### 3.5 Time-to-onset analysis

**FIGURE 5:**
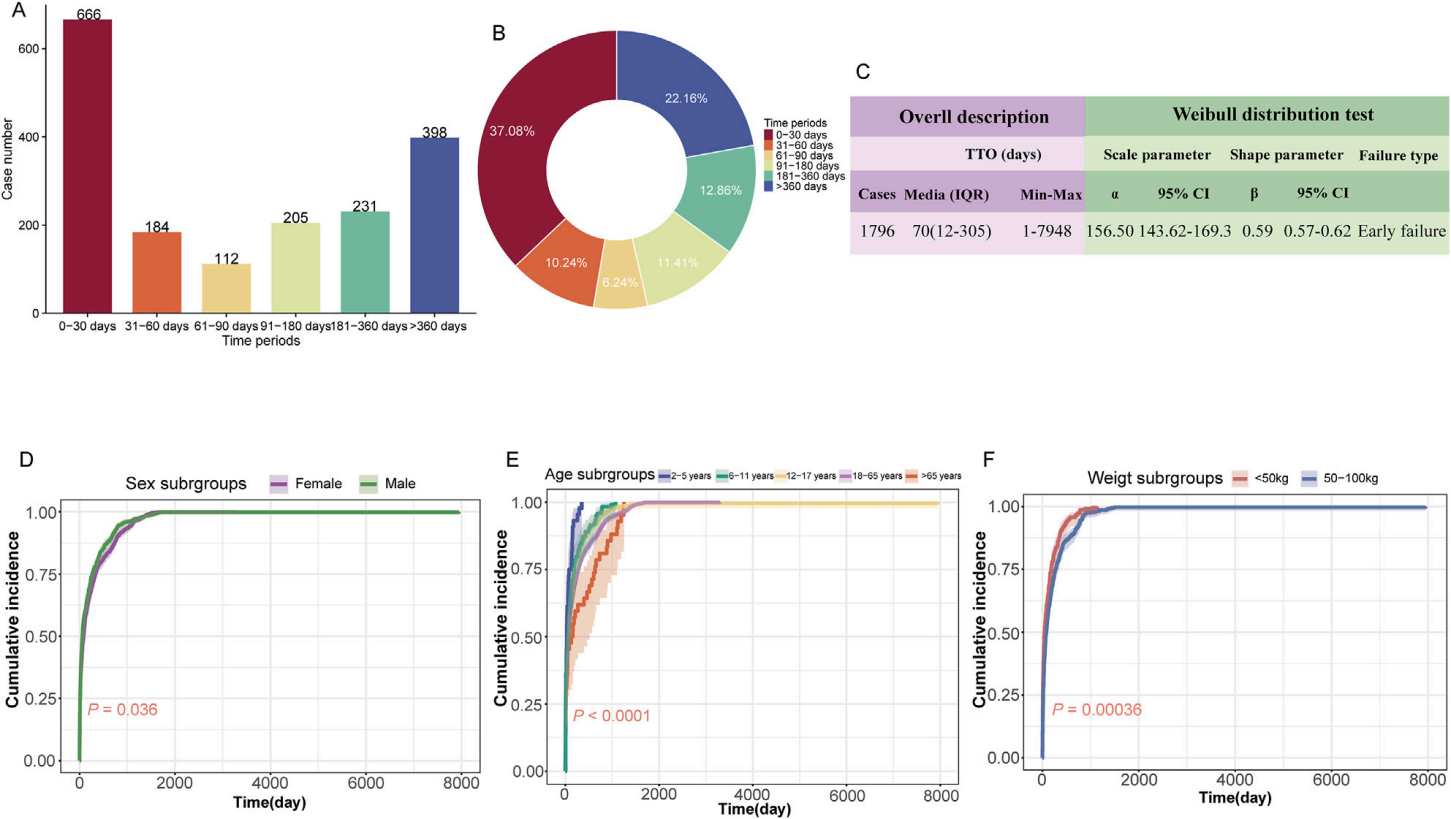
Time-to-onset (TTO) analysis of elexacaftor/tezacaftor/ivacaftor-related ADEs. **(A)** Bar charts illustrating the quantity of TTO reports across varying time intervals. **(B)** Pie charts depicting the proportion of TTO reports for different time intervals. **(C)** Weibull distribution test results for TTO analysis. The subgroup TTO analysis based on sex **(D)**, age **(E)**, and weight **(F)** was presented using Kaplan-Meier curves. IQR: interquartile range; Min: minimum; Max: maximum.

A total of 1,796 (16.0%) valid TTO reports were collected. The majority of ADEs occurred during within the first month of initiating treatment (n = 666, 37.08%), but it is worth noting that 22.16% of adverse events still occurred after 360 days (n = 398) (Figures 5A, B). The median TTO for ETI-related ADEs was 70 days, with an interquartile range (IQR) of 12–305 days. The results of the Weibull distribution test indicated an early failure type curve, suggesting that the probability of ADEs occurring decreases over time (Figure 5C). Additionally, we observed differences in the cumulative incidence of ADEs during ETI treatment across different subgroups. Notably, significant differences were observed based on sex (*P* = 0.036, Figure 5D), age (*P* < 0.0001, Figure 5E), and weight (*P* = 0.00036, Figure 5F).

**FIGURE 6:**
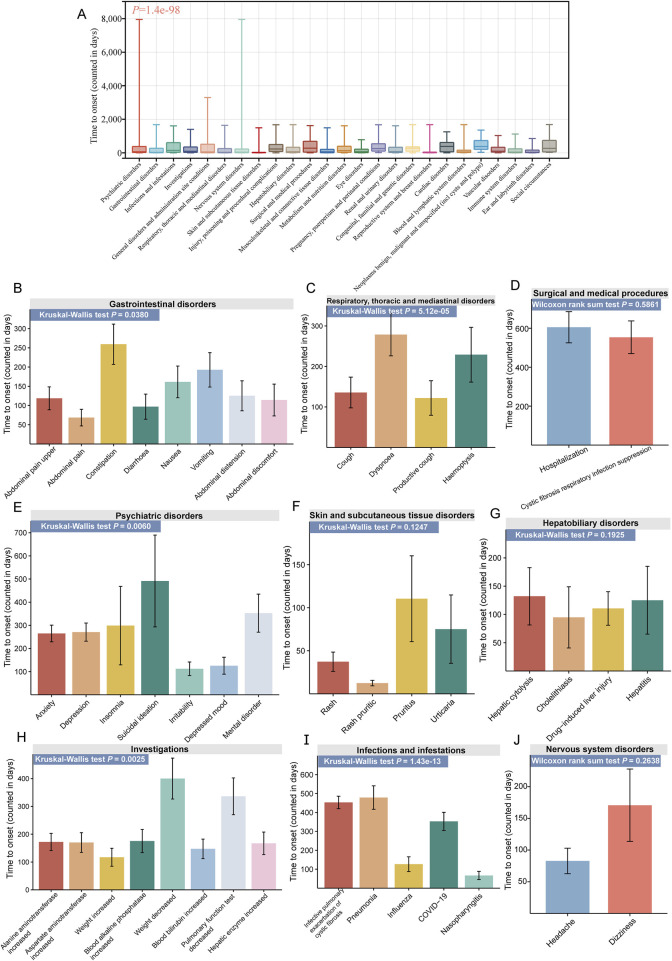
TTO analysis of ADEs at the SOC and PT levels. **(A)** Box plot of TTO at the SOC level for elexacaftor/tezacaftor/ivacaftor, with the bold line representing the median TTO; the lower end of the whisker indicates the first quartile (Q1), and the upper end indicates the third quartile (Q3). Specific comparison of TTO for PTs across nine different SOCs, including gastrointestinal disorders **(B)**, respiratory, thoracic and mediastinal disorders **(C)**, surgical and medical procedures **(D)**, psychiatric disorders **(E)**, skin and subcutaneous tissue disorders **(F)**, hepatobiliary disorders **(G)**, investigations **(H)**, infections and infestations **(I)**, and nervous system disorders **(J)**.

Figure 6 illustrates the TTO analysis of ETI-related ADEs at both the SOC and PT levels. At the SOC level, there were significant differences in the onset time of different ADEs (*P* = 1.4e-98, Figure 6A). Psychiatric disorders had the highest number of TTO reports (634 cases), with a median TTO of 68 days and an interquartile range (IQR) of 20–380 days. We also conducted TTO analysis at the PT level for ADEs under the 9 positive SOCs that met the ROR threshold (no sufficient TTO reports were collected for the other three SOCs). Significant differences in the onset time of PT were observed at the following 5 SOC levels: gastrointestinal disorders (*P* = 0.0380, Figure 6B), respiratory, thoracic and mediastinal disorders (*P* = 5.12e-05, Figure 6C), psychiatric disorders (*P* = 0.0060, Figure 6E), investigations (*P* = 0.0025, Figure 6H), and infections and infestations (*P* = 1.43e-13, Figure 6I). No such significant differences were observed in the remaining 4 SOCs: surgical and medical procedures (*P* = 0.5861, Figure 6D), skin and subcutaneous tissue disorders (*P* = 0.1247, Figure 6F), hepatobiliary disorders (*P* = 0.1925, Figure 6G), and nervous system disorders (*P* = 0.2638, Figure 6J). At the PT level, infective pulmonary exacerbation of CF had the highest number of TTO reports (169 cases), with a median TTO of 258 days and an IQR of 83–782 days. More detailed results are available in Supplementary Table S3.

## 4 Discussion

### 4.1 Demographics

Currently, a universally accepted “gold standard” for ADE signal detection has yet to be established, and disproportionality analysis remains the most common method for signal mining (Trinh et al., 2019). In this study, we performed a disproportionality analysis using the FAERS database to investigate ADEs associated with ETI. Our analysis identified both well-established ADEs listed in drug labels and previously unreported or rare ADEs, providing a comprehensive overview of ETI-related ADE reports in FAERS.

The baseline data reveal that the proportion of reports containing specific information is higher among females (49.9%) than males (37.8%), with ADE reports being most frequent among individuals aged 18–65 years (32.0%). Geographically, the United States accounted for the largest percentage of reports (77.5%). This predominance is likely attributable to the fact that the United States was the first country to approve the use of ETI, which facilitated its earlier and more widespread adoption.

Improved survival rates, with a reported median age of survival reaching up to 53.3 years, have resulted in a higher proportion of affected adults (53%) compared to children (Diab Cáceres and Zamarrón de Lucas, 2023). Notably, young females continue to exhibit poorer prognosis and median survival rates than their male counterparts, although the precise reasons for this disparity remain unclear (McIntyre, 2013). Drug-related ADEs may also be influenced by indication; in our study, over 99% of ADEs were reported in the context of CF, thereby enhancing the sensitivity of our analysis results. Historically, CF patients have experienced adverse outcomes associated with viral infections, with respiratory viral infections often identified as contributing factors to pulmonary exacerbations. It is plausible that the COVID-19 pandemic significantly impacted the increased number of reported ETI-associated ADEs in 2020 (Flume et al., 2022).

### 4.2 Known and unexpected signals

Our comprehensive pharmacovigilance analysis identified 322 positive signals associated with ETI across 22 different SOCs. Among these significant signals (Supplementary Table S1), the most frequently reported ADEs included hospitalization (960 cases), headache (702 cases), infective pulmonary exacerbation of CF (691 cases), rash (538 cases), cough (507 cases), anxiety (494 cases), and productive cough (475 cases). Additionally, we identified signals consistent with those listed in the drug label, such as cough, upper abdominal pain, weight increased, influenza, constipation, increased hepatic enzymes, acne, lower respiratory tract infection, and pruritic rash, demonstrating the reliability of our study’s findings.

Notably, upper abdominal pain (417 cases, ROR 4.94 [95% CI 4.49–5.45]) has been recognized as a potential ADE associated with ETI use, which may also be influenced by underlying conditions. A study involving 73 children and 110 adult CF patients found that 55% of children and 73% of adults experienced pain lasting more than 6 months. Additionally, 60% of children and 36% of adults reported chronic abdominal pain, with the abdomen being the most common site of chronic pain in children (Sermet-Gaudelus et al., 2009). Common causes of abdominal pain in CF patients include exocrine pancreatic insufficiency, meconium ileus, distal intestinal obstruction syndrome, constipation, and intestinal dysbiosis from frequent antibiotic use (Mainz et al., 2023). Furthermore, a case series by Safirstein et al. reported that individuals with CF experienced biliary colic shortly after initiating ETI therapy, necessitating cholecystectomy (Safirstein et al., 2021). The prevalence of abdominal pain significantly impacts the quality of life, emphasizing the importance of careful assessment and management of pain.

We also identified several unexpected ADEs associated with ETI, including anxiety (494 cases), depression (364 cases), insomnia (281 cases), nephrolithiasis (79 cases), and testicular pain (42 cases). The open-label extension study examining ETI did not report neurological or psychiatric ADEs other than headaches; however, our analysis indicated that psychiatric disorders were the most frequently reported SOC (3,566 cases) and identified as positive (ROR 2.54 [95% CI 2.45–2.63]). The common ADEs related to psychiatric disorders included anxiety, depression, mental disorder, depressed mood, and irritability. In adult CF patients, symptoms of depression are observed in 17%, while symptoms of anxiety are present in 33%, reflecting a twofold increase compared to the general population (Yohannes et al., 2012a).

Some reports describe increased symptoms of depression and anxiety in CF patients starting ETI therapy. Zhang et al. noted that there was no significant change in Patients Health Questionnaire-9 (PHQ-9) scores, with 5% of patients receiving new mental health diagnoses and 22% increasing, switching, or adding psychotropic medications, indicating potential worsening depressive symptoms (Zhang et al., 2022). The underlying mechanisms for these potential side effects remain unclear. *In vivo* studies demonstrate that ivacaftor and its metabolites act on the 5-HT2C receptor, linked to depression and anxiety, which is a target for antidepressant medications. Additionally, CFTR modulators may interfere with CYP450 enzyme metabolism, potentially reducing the effectiveness of psychiatric medications (Schneider et al., 2018).

Perinatal depression emerged as an unexpected signal with four cases and a strong signal value (ROR 13.59 [95% CI 5.06–36.51]). Perinatal depression refers to a major depressive episode occurring during pregnancy or within a year following childbirth. Approximately 15% of pregnant women experience depression, and *postpartum* depression affects about 12% of women with no prior history (Woody et al., 2017). Depression during pregnancy and *postpartum* is associated with elevated cortisol levels and diminished cortisol awakening response compared to women without these conditions (Hantsoo et al., 2023). A study by Taylor-Cousar et al. revealed that among 45 reported pregnancies exposed to ETI, clinicians suspected two maternal complications and three infant complications related to the drug (Taylor-Cousar and Jain, 2021).

The effects of CF and the potential impact of ETI on the fetus can act as psychological stressors, activating the hypothalamic-pituitary-adrenal (HPA) axis and leading to excessive cortisol release, a risk factor for depression. Further investigations are needed to assess ETI’s impact on the central nervous system. We recommend maintaining vigilance for psychological disorders following ETI use and employing validated questionnaires to monitor changes in depression and anxiety during treatment. The decision to discontinue the medication should be carefully evaluated based on the patient’s overall condition.

### 4.3 Subgroup analysis

Baseline descriptive data indicated that the proportion of ADE reports was higher in female patients compared to male patients, underscoring the importance of including sex analysis in drug safety assessments. A prospective “real world” longitudinal study found a significant increase in insomnia over time, and female participants reported more side effects than male participants (Graziano et al., 2024). To further investigate the association between sex and ADEs, a subgroup analysis was conducted, as illustrated in Supplementary Figure S1. Male patients had higher reporting ranks of insomnia, abdominal pain, and nasopharyngitis. In contrast, female patients exhibited higher associations with mental disorder, constipation and exposure during pregnancy Notably, the higher reporting rank of mental disorder (n = 107, ROR: 10.76 [95% CI 8.89–13.04]) among female reports caught our attention. Some studies suggest that females may experience greater declines in pulmonary function due to poorly controlled comorbidities, such as nutritional deficiencies, while others indicate a potential influence of sex hormones on inflammation, airway epithelial ion channel function, and bacterial phenotype modulation (Sweezey and Ratjen, 2014). Poor lung function has been associated with higher adult depression scores (Nici et al., 2006; Goldbeck et al., 2010; Yohannes et al., 2012b). Furthermore, female patients with CF have earlier bacterial colonization and more frequent lung exacerbations (Maselli et al., 2003), and the quality of life of female CF patients is lower than that of males (Sodhi et al., 2023), which has a greater impact on mood and may cause mental disorders to rank higher in female.

ETI is primarily metabolized by the hepatic enzyme CYP3A4, the most abundant isoform responsible for over 50% of known therapeutic drugs. Studies have indicated that females exhibit higher levels of CYP3A4 protein expression compared to males (Ruiz et al., 2013). Additionally, recent research has shown that estradiol positively regulates the expression of CYP3A4 mRNA, potentially leading to sex-specific variations in ETI metabolism (Choi et al., 2013). These findings emphasize the importance of considering sex-specific ADEs in clinical practice and highlight the need for personalized medication guidance to optimize the effectiveness of drug utilization.

ETI has been approved for use in patients of different ages with CF. Individuals across various age groups exhibit distinct responses to pharmacological agents, primarily in terms of drug metabolism and excretion capabilities, drug sensitivity, dosage requirements, and treatment duration. Elderly individuals frequently suffer from multiple chronic conditions and require long-term medication for various ailments. However, elderly patients with comorbidities are often excluded from clinical trials (Van Spall et al., 2007), thereby neglecting the assessment of potential drug-disease interactions in this vulnerable population (Roland, 2006). Considering the significant physiological differences between these age groups, we further performed age subgroups analysis to investigate the relationship between age and the type of drug-related ADEs. In a 3-period clinical trial, the most common ADEs observed in children with CF aged 2–5 years who took ETI were cough (46/75, 61.3%), fever (26/75, 34.7%) and rhinorrhea (25/75, 33.3%), and a 3.6-year-old child with CF and a history of behavioral problems developed severe abnormal behavior after taking ETI, including hyperactivity, aggression, urinary urgency and enuresis, leading to drug discontinuation (Wainwright et al., 2023). Our study found that, after evaluating the strength of the signals, the top three PTs in frequency among CF patients aged 2–5 years are aggression (n = 20, ROR 15.69 [95% CI 9.9–24.87]), behavior disorder (n = 13, ROR 33.23 [95% CI 18.64–59.25]), and abnormal behavior (n = 11, ROR 16.18 [95% CI 8.76–29.88]). Possible reasons for this inconsistency may include limitations of the clinical trials, differences in observer status, and the correction of signal strength. A randomized, double-blind, placebo-controlled Phase 3b trial was conducted to evaluate the efficacy and safety of ETI in children aged 6–11 years with F508del CF heterozygosity and minimal functional CFTR mutations (F/MF genotype). It was found that the most common AEs in the ETI group were headache (18/60, 30%) and cough (14/60, 23.3%) (Mall et al., 2022). Another evaluation of the effectiveness and safety of ETI treatment in children with CF aged 6–11 years in a real-world setting found that pulmonary exacerbations (57 episodes in 26 children, 76.5%), self-limited rash (17.6% in 6 children), and acute otitis media (8.8% in 3 children) were the three most common ADEs (Daccò et al., 2024). However, anxiety (n = 28, ROR 6.98 [95% CI 4.76–10.22]) and headache (n = 24, ROR 3.04 [95% CI 2.02–4.57]) were top two PTs among the CF patients aged 6–11 years observed in our study. In addition, our research found that intracranial pressure increased (n = 14, ROR 24.48 [95% CI 14.02–42.75]) in reporters aged 6–11, which was also observed in another clinical trial (Wainwright et al., 2023). It is worth noting that we found CF patients aged 12–17 years who received ETI treatment had a higher reporting rank of suicidal ideation (n = 26, ROR 3.8 [95% CI 2.57–5.63]) compared to other age subgroups. Previous studies have shown that 12- to 17-year-old adolescents with CF have significantly increased healthcare resource utilization and costs, i.e., increased frequency of hospital, outpatient, and emergency department visits, increased need for medications, and increased psychological burden associated with the disease (Thorat et al., 2021). A high incidence of suicidal ideation has been reported in CF patients, with up to 11% in adults and even higher (up to 22%) in adolescents, although a causal relationship with ETI treatment remains uncertain (Ramsey et al., 2024). Our results still suggest the monitoring of key populations and early intervention for patients. In term of reporters aged 18–65 years, our study found abdominal pain (n = 107, ROR 2.44 [95% CI 2.01–2.95]) and constipation (n = 102, ROR 3.57 [95% CI 2.93–4.34]) to be the top 15 PTs that were ranked differently from other age subgroups. Although gastrointestinal symptoms in adults with CF improve significantly during the first 1.5 years of treatment with ETI, they seem to diminish with long-term use (Caley et al., 2025). It needs to be considered whether the efficacy of the drug diminishes or whether the side effects are causing it. Headache was a signal that occurred in all subgroups, so the management of headaches can help improve the medication experience for people of different ages. Finally, due to the limited number of reporters at each stage, our results can only offer partial reference. However, this approach is necessary as the long-term use of medications is accompanied by the patient’s increasing age, making it crucial to identify specific and common ADEs across different age groups. This aspect should be considered in future studies.

### 4.4 Time to onset analysis

Our analyses revealed that the TTO of ADEs associated with ETI exhibited an early failure pattern, indicating a higher likelihood of ADEs shortly after treatment initiation. The median TTO for ETI-associated ADEs was 70 days, with the majority occurring within the first month (666 cases, 37.08%). Notably, a significant number of ADEs (398 cases, 22.16%) can still manifest after a year of treatment. Psychiatric disorders were the most frequently reported ADEs, comprising 634 cases with a median TTO of 68 days, followed by gastrointestinal disorders with 536 cases and a median TTO of 28 days.

Another significant finding from our study is the statistical difference in the cumulative incidence of ADEs across different age subgroups. Within the same cumulative incidence, ADEs occurred earlier in younger patients (2–5 years and 6–11 years subgroups) compared to older groups. Considering that younger patients may have a higher metabolic rate and thus faster drug processing, the onset of ADEs occur earlier, whereas the latency period for ADEs in adult patients may be longer (Mangoni and Jackson, 2004). We also observed statistical differences in the cumulative incidence of ADEs across sex and weight subgroups. Within the same cumulative incidence, males experienced ADEs earlier than females, and patients with lower body weight had earlier onset of ADEs compared to those with moderate body weight. Aside from the potential influence of hormone levels (e.g., testosterone), males may have a higher drug metabolism rate or different drug absorption pattern, leading to an earlier occurrence of drug-related ADEs (Zuern et al., 2009). Lower body weight patients may have relatively higher drug concentrations and less capacity for drug storage, which could result in earlier onset of drug-related ADEs (Brill et al., 2012). However, given the low reporting rate of TTO (16.0%), these findings should be interpreted with caution.

Within gastrointestinal disorders, abdominal pain had the earliest onset (median TTO of 7 days), while distal intestinal obstruction syndrome had the latest onset (111 days). For psychiatric symptoms, insomnia occurred earlier (median TTO of 40 days), whereas suicidal ideation manifested later (187 days). These findings highlight the importance of rigorous monitoring and proactive management of ADEs, providing a valuable resource for managing patients undergoing ETI treatment.

### 4.5 Limitations

Although this study provides reliable scientific evidence for the safety evaluation of ETI from multiple perspectives, several limitations still exist. First, as a self-reporting system, the FAERS database may be subject to inherent issues such as report omissions, delayed reporting, inconsistent report quality, and reporting bias (Zhou and Yao, 2024). Second, the lack of detailed clinical information about patients, such as comorbidities, underlying diseases, and medication history (e.g., concomitant drug use), could potentially affect the stability of the results due to these confounding factors (Zhang Y. et al., 2024). Moreover, the analysis of disproportionality data is limited to assessing the strength of adverse event signals and does not allow for the quantification of risk or the determination of causal relationships with the drug (Wei et al., 2024). Finally, a key limitation of our study lies in the signal detection process. In this analysis, adverse events related to ETI were compared with those associated with all other drugs in the FAERS database, which may inadvertently lead to the identification of nonspecific signals that overlap with symptoms of cystic fibrosis itself (Jiang et al., 2025). Given these limitations, caution is required when interpreting our findings, and further clinical evaluations are essential to validate these associations. Nevertheless, this comprehensive analysis lays a solid foundation for future research on ETI.

## 5 Conclusion

This study analyzed 28,366 ADEs linked to ETI using the FAERS database. Significant findings included positive signals for psychiatric disorders such as anxiety and depression, as well as unexpected signals such as insomnia, and nephrolithiasis. Most ADEs occurred shortly after treatment initiation, with some occurring more than year after treatment, emphasizing the need for continuous monitoring. Significant sex-specific differences were observed, with females exhibiting a higher reporting rank for mental disorder and constipation, while males reported higher ranks of insomnia and nasopharyngitis. Limitations of the study include potential reporting biases and the inability to establish causality. These findings underscore the necessity for personalized management strategies and highlight the need for further research to elucidate the mechanisms underlying these ADEs, balancing the benefits of ETI with appropriate risk management.

## Data Availability

The original contributions presented in the study are included in the article/Supplementary Material, further inquiries can be directed to the corresponding author.
